# Exploring Aromaticity Effects on Electronic Transport in Cyclo[n]carbon Single-Molecule Junctions

**DOI:** 10.3390/molecules29163827

**Published:** 2024-08-12

**Authors:** Peiqi Yang, Haoyang Pan, Yudi Wang, Jie Li, Yangyu Dong, Yongfeng Wang, Shimin Hou

**Affiliations:** 1 Key Laboratory for the Physics and Chemistry of Nanodevices, School of Electronics, Peking University, Beijing 100871, China; peggyyang@stu.pku.edu.cn (P.Y.); 1801111578@pku.edu.cn (H.P.); ydwang@pku.edu.cn (Y.W.); lijie0120@stu.pku.edu.cn (J.L.); 2101111880@stu.pku.edu.cn (Y.D.); yongfengwang@pku.edu.cn (Y.W.); 2 Institute of Spin Science and Technology, South China University of Technology, Guangzhou 511442, China; 3 Centre for Nanoscale Science and Technology, Academy for Advanced Interdisciplinary Studies, Peking University, Beijing 100871, China

**Keywords:** cyclo[n]carbon, molecular junction, low-bias conductance, aromaticity

## Abstract

Cyclo[n]carbon (C_n_) is one member of the all-carbon allotrope family with potential applications in next-generation electronic devices. By employing first-principles quantum transport calculations, we have investigated the electronic transport properties of single-molecule junctions of C_n_, with n = 14, 16, 18, and 20, connected to two bulk gold electrodes, uncovering notable distinctions arising from the varying aromaticities. For the doubly aromatic C_14_ and C_18_ molecules, slightly deformed complexes at the singlet state arise after bonding with one Au atom at each side; in contrast, the reduced energy gaps between the highest occupied and the lowest unoccupied molecular orbitals due to the orbital reordering observed in the doubly anti-aromatic C_16_ and C_20_ molecules lead to heavily deformed asymmetric complexes at the triplet state. Consequently, spin-unpolarized transmission functions are obtained for the Au-C_14/18_-Au junctions, while spin-polarized transmission appears in the Au-C_16/20_-Au junctions. Furthermore, the asymmetric in-plane π-type hybrid molecular orbitals of the Au-C_16/20_-Au junctions contribute to two broad but low transmission peaks far away from the Fermi level (*E*_f_), while the out-of-plane π-type hybrid molecular orbitals dominate two sharp transmission peaks that are adjacent to *E*_f_, thus resulting in much lower transmission coefficients at *E*_f_ compared to those of the Au-C_14/18_-Au junctions. Our findings are helpful for the design and application of future cyclo[n]carbon-based molecular electronic devices.

## 1. Introduction

All-carbon allotropes such as graphene [1], fullerenes [2], and carbon nanotubes [3] have attracted great attention for their outstanding physicochemical properties and potential applications in next-generation technologies. Cyclo[n]carbons (C_n_) are ring-like structures of all-carbon allotropes composed of two coordinated sp-hybridized carbon atoms. While early experiments have confirmed the existence of cyclo[n]carbons in the gas phase [4,5,6,7], their instability has hindered their real-space characterization until recently, whereby bond-resolved atomic force microscopy (AFM) images of C_18_ on the NaCl surface [8] revealed its polyynic structure, followed by C_10_, C_12_, C_14_, C_16_, and then C_20_ [9,10,11]. Their successful synthesis has spurred extensive theoretical studies into various aspects of C_n_, including their geometry [12,13,14,15], aromaticity [16,17,18,19], electronic structure [14,20,21], bonding character [21], electric field effect [14,22], optical properties [14,23], carbon tunneling [24], and surface coupling [20,25]. Applications such as catalysts [26] and spintronic devices [27,28] have also been explored.

Understanding how electrons propagate through C_n_ is essential for their practical applications in electronic devices. Molecular junctions consisting of a single or a few molecules sandwiched between two electrodes provide a platform for studying the electronic transport properties at the molecular scale. Zhang et al. reported current–voltage (I–V) characteristics of C_18_ molecular junctions with one-dimensional (1D) carbon chain electrodes, two-dimensional (2D) graphene electrodes, and three-dimensional (3D) bulk silver electrodes; however, they neglected the atomic changes of C_18_ possibly brought by their contact with the electrodes [29]. Other studies have focused on the I–V characteristics like negative differential resistance [30], or spintronic transport characteristics [27,28,31] like the spin filtering effect and the magnetoresistance of C_18_ sandwiched between graphene nanoribbons. The electronic transport properties of other C_n_ remain unexplored, particularly in junctions formed with conventional metal electrodes like gold. Moreover, previous studies have demonstrated the variations in the electronic structures of isolated C_n_ based on their aromaticities [9,16,17,18]; therefore, how the aromaticity affects the electronic propagation is very intriguing. 

Herein, we present a systematic study of the electronic transport properties of C_n_ (n = 14, 16, 18, 20) molecular junctions with gold electrodes using the non-equilibrium Green’s function formalism combined with density functional theory (that is, the NEGF + DFT approach) [32,33,34,35,36,37,38,39]. Our calculations reveal distinct junction geometries and spin states resulting from different C_n_ aromaticities, which lead to significantly different electronic transport performances between the doubly aromatic C_14/18_ and the doubly anti-aromatic C_16/20_ after connecting to the gold electrodes. Furthermore, we extend our calculations to larger Au-C_n_-Au junctions involving more carbon atoms. 

## 2. Results and Discussion

**Figure 1:**
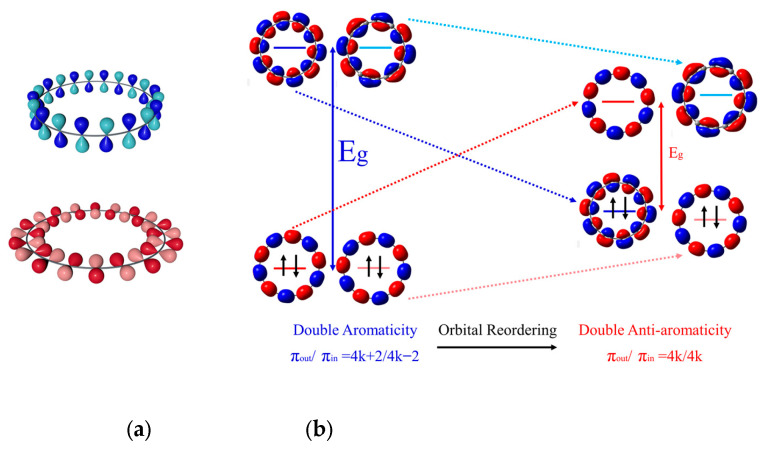
(**a**) Visualizations of the π_out_ (upper) and π_in_ MOs (bottom) of the C_20_ molecule. (**b**) The orbital reordering process in the anti-aromatic C_20_ molecule.

The optimized atomic structures of cyclo[n]carbons (n = 14, 16, 18, 20) at the ωB97XD/6-311+G(d,p) level are displayed in Figure S1. Our calculations indicate that C_14_ is an intermediate with a tiny bond length alternation (BLA) of 0.047 Å and a large bond angle alternation (BAA) of 25.67°, possessing a C_7h_ symmetry; in contrast, C_16_, C_18_, and C_20_, which are, respectively, of C_8h_, D_9h_, and C_10h_ symmetries, exhibit polyynic structures consisting of alternating triple and single bonds with a much larger BLA (0.15 Å, 0.12 Å, and 0.14Å, respectively) and nearly zero or no BAA (0.70°, 0, and 0.14°, respectively). The less symmetric atomic structures of C_n_ with n = 16, 18, and 20 may result from the symmetry breaking event, a consequence of the second-order Jahn–Teller effect [13]. Our calculations agree well with previous experimental observations [8,9,10,11] and theoretical calculations applying HF (Hartree–Fock) [40], CCSD (coupled-cluster singles and doubles) [21,41], ab initio CASSCF (complete active space self-consistent field) [20], and DFT [12,14,16,17,20,21,26]. Despite sharing a similar ring structure with benzene, these C_n_ molecules possess two types of orthogonal π MOs. One is the out-of-plane π (π_out_) MOs (see the upper panel of Figure 1a), and the other is the in-plane π (π_in_) MOs (see the bottom panel of Figure 1a). These two types of π orbitals are separately occupied by electrons [17]. Consequently, depending on the occupancy of these two types of π MOs, C_n_ can exhibit double aromaticity, double anti-aromaticity, or conflicting aromaticity [42].

The frontier molecular orbitals (FMOs) and their corresponding energies of C_n_ (n = 14, 16, 18, 20) are presented in Figure S1. Our calculations suggest C_14/18_ is doubly aromatic, containing 14/18 (4k + 2) π_in_ and π_out_ electrons. In contrast, C_16/20_ is doubly anti-aromatic, since it has 16/20 (4k) π_in_ and π_out_ electrons. Furthermore, upon closer examination of their FMOs and energies, we observe that for the doubly aromatic C_14/18_, π orbitals appear as degenerate or nearly degenerate pairs of the same orientation and semblable structures distinguished by rotation. For instance, the HOMO and HOMO−1 orbitals of C_18_ are degenerate π_in_ orbitals. However, for the doubly anti-aromatic C_16/20_, obeying this rule will result in the occupancy of the two types of π orbitals by 4k + 2/4k − 2 electrons. Therefore, in order to preserve the double anti-aromaticity, orbital reordering occurs where one of the π_out_ orbitals pairs with one of the π_in_ orbitals as HOMO−1 and HOMO, while the other π_out_ orbital related to the rotation pairs with the left π_in_ orbital as LUMO and LUMO + 1 (see the orbital reordering process in C_20_, as shown in Figure 1b). This orbital reordering is likely to reduce the HOMO–LUMO gaps of the doubly anti-aromatic molecules. Indeed, the HOMO–LUMO gaps of the doubly anti-aromatic C_16/20_ are, respectively, calculated to be 5.87 eV and 5.67 eV, which are much smaller compared to those of the doubly aromatic C_14/18_, which are 7.14 eV and 6.76 eV. 

**Figure 2:**
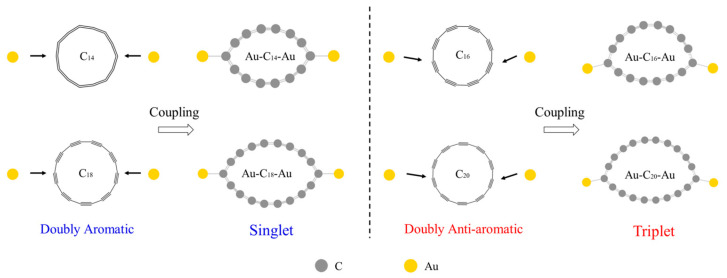
The atomic structures for the most probable Au-C_n_-Au (n = 14, 16, 18, 20) complexes and their spin states appearing in the corresponding molecular junctions. The arrows point to the C atoms that are bonded with the Au atoms after coupling.

Having illustrated the atomic and electronic structures of the isolated C_n_ molecules, we now move on to explore the electronic transport characteristics of the Au-C_n_-Au molecular junctions. We construct the junctions by attaching the C_n_ molecules to the gold electrodes through one gold adatom on both sides without any other anchor groups, because it has been experimentally demonstrated that carbon atoms with radially oriented π orbitals can directly bond to the gold electrodes [43,44]. Before probing the junction transport properties, we first explore the interactions between the C_n_ molecules and Au atoms. Hence, we construct isolated Au-C_n_-Au complexes to examine the atomic and electronic changes in the C_n_ molecules after bonding to the Au atoms. The structures at both the singlet and triplet states can be obtained. By comparing the total energy of the optimized structures and considering the possibility of the C_n_ molecules bonding with the gold electrodes, we identify the most probable Au-C_n_-Au (n = 14, 16, 18, 20) configurations appearing in the junctions (see Figure 2). Large Au-C bonding energies are obtained, of 1.60 eV, 1.45 eV, 1.39 eV, and 1.29 eV for n = 14, 16, 18, and 20, respectively. This suggests the formation of strong covalent Au-C bonds [45], and also results in severe deformations of C_n_. A similar deformation has also been reported for the C_18_ molecular junction with graphene nanoribbon electrodes [31,46]. Meanwhile, clear distinctions between the doubly aromatic and doubly anti-aromatic C_n_ molecules are observed after bonding with the Au atoms, including the more asymmetrical atomic structure and the spin-polarized triplet state for the Au-C_16/20_-Au complexes. We attribute this difference to the lower HOMO–LUMO gaps of the doubly anti-aromatic C_16/20_ molecules, which are easier to overcome via the exchange energy acting on electrons with the same spin, favoring a structure at a triplet state after bonding with the Au atoms.

**Figure 3:**
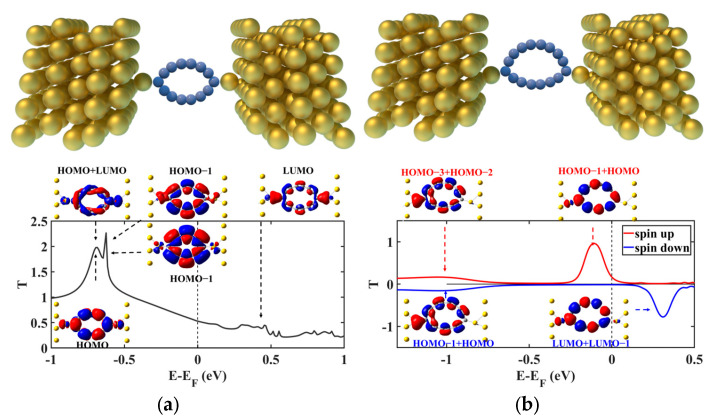
The atomic junction structures (upper panel) and the corresponding equilibrium transmission spectra (bottom panel) of the Au-C_14_-Au (**a**) and Au-C_16_-Au (**b**) molecular junctions, together with the conducting eigenchannels for selected transmission peaks.

Then, we construct the junction structures with the obtained Au-C_n_-Au complexes, which are shown in the upper panels of Figure 3 and Figure S2. And the corresponding equilibrium transmission functions around the Fermi Level (*E*_f_) are also plotted in the bottom panels of Figure 3 and Figure S2, with *E*_f_ being set to zero. The transmission coefficients at *E*_f_ (*T*(*E*_f_)) are, respectively, calculated to be 0.53, 0.18 (spin-up)/0.01 (spin-down), 0.50, and 0.16 (spin-up)/0.01(spin-down) for the C_14_, C_16_, C_18_, and C_20_ junctions. Remarkable spin-polarized features with a much smaller *T*(*E*_f_) are observed for the Au-C_16/20_-Au junctions compared to the Au-C_14/18_-Au junctions. This observation is somewhat perplexing because the doubly anti-aromatic molecules are expected to exhibit a higher *T*(*E*_f_) due to their smaller HOMO–LUMO gaps. Attempting to elucidate this peculiar phenomenon, conducting eigenchannels were calculated to identify the dominating MOs (depicted in Figure 3 and Figure S2) [47].

**Figure 4:**
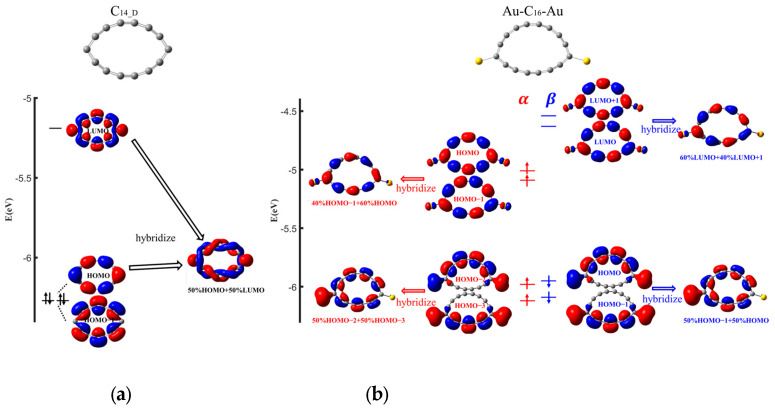
FMOs and the hybrid MOs of the isolated C_14_D_ molecule (**a**) and the Au-C_16_-Au complex (**b**).

For the doubly aromatic C_14/18_, spin-unpolarized and highly similar transmission functions are obtained for their single-molecule junctions with Au electrodes, which display two apparent resonance peaks below *E*_f_ (see Figure 3a and Figure S2a). In the case of the Au-C_14_-Au junction, the transmission peaks located at −0.69 eV and −0.65 eV can be decomposed into multiple eigenchannels, resulting in their heights exceeding unity. Due to the strong coupling between C_n_ and the two Au adatoms, MO switching happens, and some MOs heavily hybridize with each other or with some orbitals of the Au adatoms, generating new orbitals contributing to the junction transmission. The shapes of the eigenchannels suggest that after connecting to the gold electrodes, HOMO−1 and HOMO exchange their energetic order. The HOMO and LUMO of the isolated deformed C_14_ molecule (C_14_D_) hybridize and contribute to one eigenchannel of the peak centered at −0.69 eV (see FMOs and their hybrid shown in Figure 4a), along with the other one, which is dominated by the HOMO. Moreover, besides the one eigenchannel contributed by the HOMO, the HOMO−1 of C_14_D_ combining with the 6s and 5d*_z2_*, 5d*_yz_* orbitals of the Au adatoms forms two new orbitals contributing to the peak at −0.65 eV. And the sharpness of this peak arises from the nodes of the HOMO−1 at the C atoms bonded to the Au adatoms, leading to reduced coupling. Despite the large transmission coefficients of these two peaks, they decay rapidly towards *E*_f_ and thus contribute negligibly to the transmission around *E*_f_. In contrast, the LUMO of C_14_D_, which exhibits a much stronger coupling with the 6s orbitals of the Au adatoms due to its π_in_ character, results in a broadened and steady transmission between −0.5 eV and 1 eV and thus dominates *T*(*E*_f_). The overall shape of the transmission spectrum of the Au-C_18_-Au molecular junction is similar to that of the Au-C_14_-Au junction (see the dominating MOs of the isolated deformed C_18_ molecule (C_18_D_) contributing to the transmission peaks shown in Figures S2a and S3a), except that the two transmission peaks change their places because of the different coupling effects between C_18_D_ and C_14_D_ with the Au electrodes; additionally, they also move towards *E*_f_ due to the smaller HOMO–LUMO gap of C_18_D_. 

For the doubly anti-aromatic C_16/20_, the difference between the spin-up and spin-down population of the C_n_ molecules in the junctions is calculated to be 1.53, leading to remarkable spin-polarized transmission functions (see Figure 3b and Figure S2b). This spin-unpolarized to spin-polarized transition of the C_16/20_ molecules indicates their much stronger electronic couplings with the Au adatoms so that the FMOs of the isolated Au-C_16/20_-Au complexes are employed to analyze the dominating MOs of the transmission peaks (see Figure 4b and Figure S3b). The transmission function of the Au-C_16_-Au junction reveals two prominent peaks for both the spin-up and spin-down channels located at −1 eV and −0.11 eV (spin-up)/0.29 eV (spin-down). For the spin-up channel, the peak away from *E*_f_ is dominated by one hybrid MO composed of the HOMO−3 and HOMO−2 orbitals of the isolated Au-C_16_-Au complex. While its π_in_ character exhibits a significant coupling to one gold electrode, leading to the broadening of the peak, its asymmetric nature also results in a much lower peak height. Inspecting the HOMO−3 and HOMO−2 orbitals, we can find that these two MOs exhibit similar orbital shape patterns and adjacent energies. This observation suggests that a potential rule for MO hybridization may exist for these doubly anti-aromatic C_n_ molecules in the junctions. The spin-up HOMO−1 and HOMO orbitals are another pair that satisfies this criterion; indeed, they hybridize and dominate the transmission peak centered at −0.11 eV. However, as this π_out_ MO has less asymmetry, this hybrid MO contributes to the relatively higher peak decaying rapidly towards *E*_f_. Likewise, for the spin-down channel, the asymmetric hybrid π_in_ MO formed by HOMO−1 and HOMO dominates the broadened yet low peak centered at −1 eV, while the less asymmetric hybrid π_out_ MO composed of the LUMO and LUMO + 1 dominates the relatively high but sharp peak located at 0.29 eV. Therefore, although both the spin-up and spin-down resonance peaks are much closer to *E*_f_, their asymmetric π_out_ nature makes them decay quickly towards *E*_f_, thus the total transmission at *E*_f_ is still less than that of the Au-C_14/18_-Au junctions. The overall shape of the transmission function of the Au-C_20_-Au molecular junction is nearly identical to that of the Au-C_16_-Au junction, as shown in Figures S2b and S3b.

Finally, we further explore the electronic transport properties of larger Au-C_n_-Au junctions containing more carbon atoms (n = 22, 24, 26, 28). For the doubly aromatic C_22/26_, they form slightly deformed complexes at the singlet state after bonding with two Au atoms like C_14/18_, and their molecular junctions share similar spin-unpolarized transmission functions with the Au-C_14/18_-Au junctions, possessing a large *T*(*E*_f_) (see Figure S4a,c). Meanwhile, the reduced HOMO–LUMO gaps of the doubly anti-aromatic C_24/28_ molecules lead to heavily deformed and asymmetric complexes at the triplet state after bonding to two Au atoms, so that their molecular junctions have spin-polarized transmission functions and a small *T*(*E*_f_), resembling the transport characteristics observed in the Au-C_16/20_-Au junctions (see Figure S4b,d).

## 3. Computational Methods

Geometry optimization and electronic structure calculations of the isolated C_n_ and Au-C_n_-Au complexes were carried out by employing the Gaussian16 DFT package with the range-separated hybrid functional ωB97XD [48], which has been proven to provide the correct qualitative polyynic structure of cyclo[n]carbons (n > 14) [11,16,20,21]. The 6-311+G(d,p) basis set is employed for carbon atoms; the SDD basis set is employed for gold atoms [49,50,51]. Frequency analysis is performed to verify the nature of the stationary points. For comparison, the generalized gradient approximation (GGA) within the Perdew-Burke-Ernzerhof (PBE) formulation [52] is also used for calculating the molecular orbitals (MOs) of the Au-C_n_-Au complexes and the deformed C_n_ molecules in the gas phase.

Quantum transport calculations were performed using TRANSISESTA code, which is a practical implementation of the NEGF + DFT approach, employing SIESTA as the DFT engine [53,54,55]. The atom cores are described using improved Troullier–Martins norm-conserving pseudopotentials (PPs), and the wave functions of the valence electrons are expanded over a finite-range numerical orbital basis set [56]. The default double-zeta plus polarization (DZP) basis set is used for Au atoms and a user-defined DZP is constructed for C atoms. The PBE GGA is used as the approximate exchange-correlation functional. Although GGA, like PBE, often underestimates the energy gap between the highest occupied molecular orbital (HOMO) and the lowest unoccupied molecular orbital (LUMO) since it is unable to describe the derivative discontinuity of the DFT potential [57], we have compared the MOs of the Au-C_n_-Au complexes and the deformed C_n_ molecules at both the ωb97XD and PBE levels (see Figure S5); their similar MO shapes and energy orders suggest that our calculations are able to qualitatively describe the transport properties of the Au-C_n_-Au molecular junctions. The unit cell of the extended molecule is composed of the central C_n_ molecule, the gold adatoms, and ten Au (111) atomic layers with a 4 × 5 in-plane supercell. The C_n_ molecule is placed in the *y-z* plane, and the transport is defined along the *z*-axis. We always consider periodic boundary conditions in the plane transverse to the transport. An equivalent energy cutoff of 200.0 Ry is taken for the real-space mesh, and the Brillouin zone is sampled using a 4 × 4 × 1 *k*-point mesh. The spin-polarized transmission function $T_{\sigma }(E)$ for the spin-up and spin-down electrons (σ=↑/↓) is evaluated as follows:

(1) $$T_{\sigma }\left(E\right)=\frac{1}{\Omega_{2DBZ}}\int_{2DBZ}T_{\sigma }(\vec{k},E)d\vec{k}$$

where $\Omega_{2DBZ}$ is the area of the two-dimensional Brillouin zone (*2DBZ*) that is orthogonal to the transport direction. The *k*-dependent transmission coefficient $T_{\sigma }(\vec{k};E)$ is defined as follows:

(2) $$T_{\sigma }\left(\vec{k},E\right)=Tr[{{\Gamma_{L,\sigma }G}_{M,\sigma }^{r}\Gamma_{R,\sigma }G}_{M,\sigma }^{r+}]$$

where $G_{M,\sigma }^{r}$ is the retarded Green’s function matrix of the extended molecule, and $\Gamma_{L/R,\;\sigma }$ represents the broadening function matrix describing the interaction between the left/right electrode and the extended molecule. In a spin-unpolarized junction, the same transmission functions are obtained for both the spin-up and spin-down channels.

## 4. Conclusions

We have systemically investigated the electronic transport properties of cyclo[n]carbon (n = 14, 16, 18, 20) single-molecule junctions connected to bulk gold electrodes by employing the NEGF+DFT approach. Our calculations show that the isolated C_14_ and C_18_ molecules exhibit double aromaticity, while the C_16_ and C_20_ molecules in the gas phase display double anti-aromaticity with a smaller HOMO–LUMO gap associated with orbital reordering. The bonding to Au atoms induces the deformation of the C_n_, especially for the doubly anti-aromatic C_16/20_ molecules for which the corresponding more asymmetric complexes are in the triplet state due to their reduced HOMO–LUMO gaps. Distinguished spin states and junction geometries originating from different aromaticities lead to significantly different transport properties between these doubly aromatic and anti-aromatic C_n_ molecules when they form Au-C_n_-Au single-molecule junctions. In detail, the Au-C_14/18_-Au junctions demonstrate a spin-unpolarized transport with a relatively large transmission around *E*_f_. In contrast, spin-polarized transmission occurs in the Au-C_16/20_-Au molecular junctions, and the asymmetric hybrid π_in_ MOs contribute to two broad but low peaks well below *E*_f_, while the two peaks adjacent to *E*_f_ are dominated by hybrid π_out_ MOs decaying fast towards *E*_f_, which result in a much smaller *T*(*E*_f_). We further extend our studies to larger junctions with more carbon atoms, and obtain very similar transport characteristics for cyclo[n]carbons with n = 22, 24, 26, and 28. These findings are beneficial to design and construct molecular electronic devices with cyclo[n]carbon molecules.

## Data Availability

Data are available from the corresponding author on request.

## Supplementary Materials

The following supporting information can be downloaded at: https://www.mdpi.com/article/10.3390/molecules29163827/s1, Figure S1: Optimized atomic structures and FMOs of the isolated C_n_ molecules with n = 14, 16, 18, and 20 calculated at the ωB97XD/6-311+G(d, p) level; Figure S2: The atomic structures (upper panel) and the corresponding equilibrium transmission spectra (bottom panel) of the Au-C_18_-Au (a) and Au-C_20_-Au (b) molecular junctions, together with the conducting eigenchannels for selected transmission peaks; Figure S3: FMOs and the hybrid MOs of the isolated C_18_D_ molecule (a) and the Au-C_20_-Au complex (b); Figure S4: The equilibrium transmission spectra of the Au-C_22_-Au (a), Au-C_24_-Au (b), Au-C_26_-Au (c) and Au-C_28_-Au (d) molecular junctions are displayed in the left panels, and their corresponding atomic structures are shown in the right panels; Figure S5: FMOs of the isolated C_14_D/18_D_ molecules (a) and the Au-C_16/20_-Au complexes (b) calculated at both the ωB97XD and PBE levels; Cartesian coordinates of the Au-C_n_-Au molecular junctions (unit: Å).
