# Supplementary material for: Exploring Aromaticity Effects on Electronic Transport in Cyclo[n]carbon Single-Molecule Junctions
Source: Molecules. 2024 Aug 12;29(16):3827. doi: 10.3390/molecules29163827 (PMC11356915; doi:10.3390/molecules29163827)
Supplement: Supplementary file 1 [file molecules-29-03827-s001.zip › molecules-3096557-supplementary.docx]

Exploring Aromaticity Effects on Electronic Transport in
Cyclo[n]carbon Single-Molecule Junctions

Peiqi Yang ^1^, Haoyang Pan ^1,2^, Yudi Wang ^1^, Jie Li ^1^, Yangyu Dong ^1,3^, Yongfeng Wang ^1^ and Shimin Hou ^1,3,^*

^1^ Key Laboratory for the Physics and Chemistry of Nanodevices, School of Electronics, Peking University, Beijing 100871, China

^2^ Institute of Spin Science and Technology, South China University of Technology, Guangzhou 511442, China

^3^ Centre for Nanoscale Science and Technology, Academy for Advanced Interdisciplinary Studies,
Peking University, Beijing 100871, China

***** Correspondence: smhou@pku.edu.cn


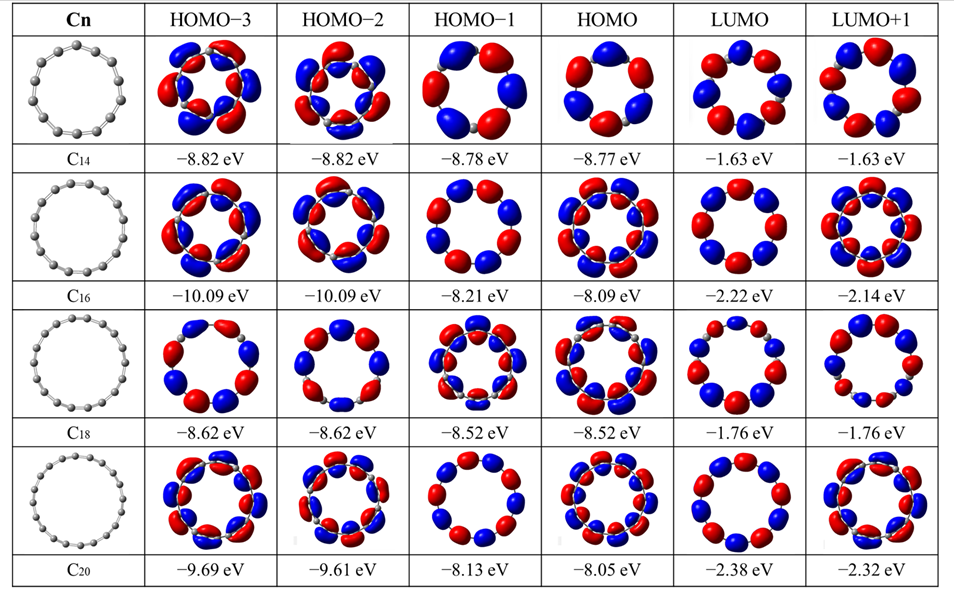


**Figure S1.** Optimized atomic structures and FMOs of the isolated C_n_ molecules with n= 14, 16, 18, and 20 calculated at the ωB97XD/6-311+G(d,p) level.

**
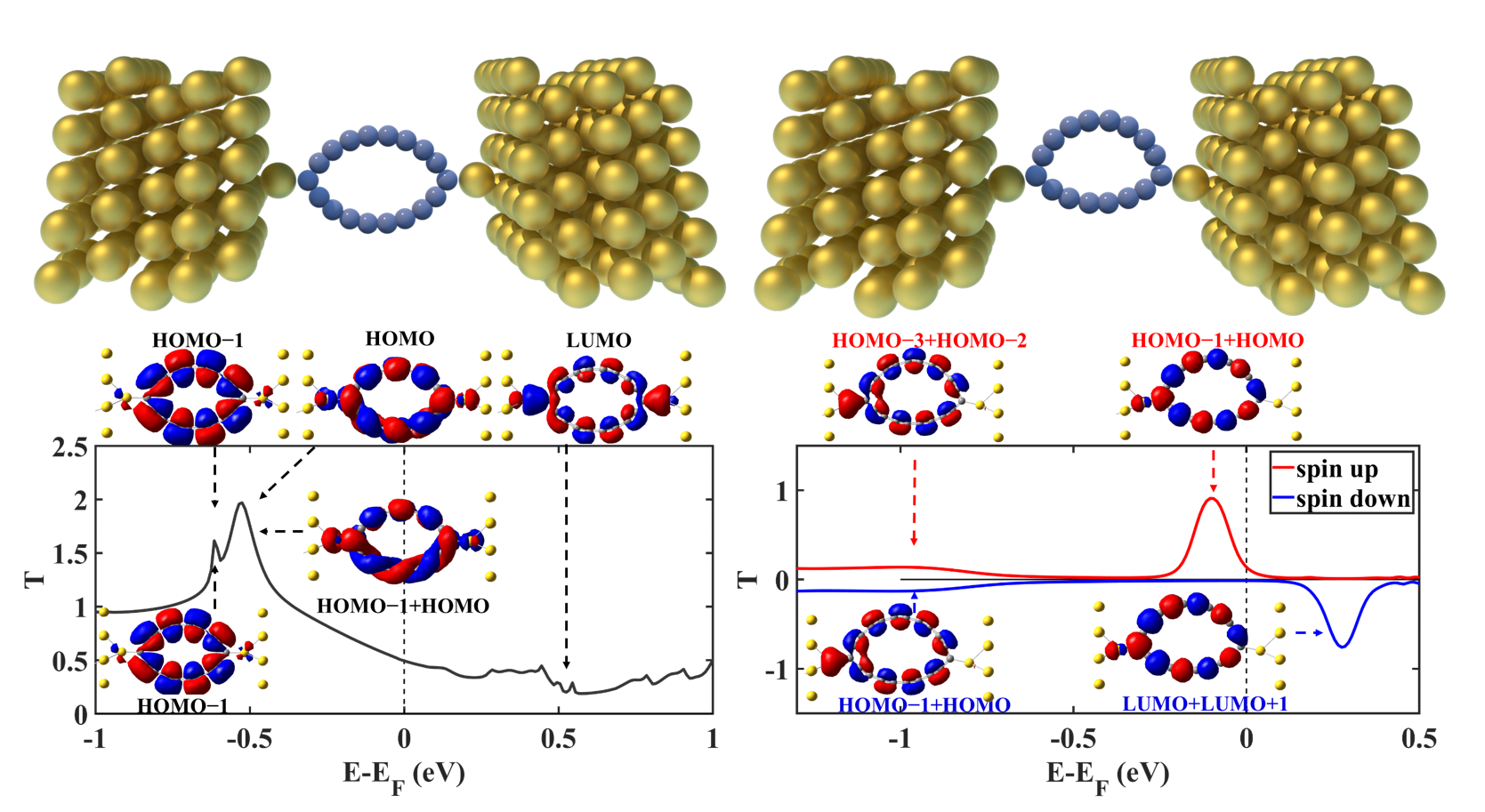
**

(**a**) (**b**)

**Figure S2.** The atomic structures (upper panel) and the corresponding equilibrium transmission spectra (bottom panel) of the Au-C_18_-Au (**a**) and Au-C_20_-Au (**b**) molecular junctions, together with the conducting eigenchannels for selected transmission peaks.


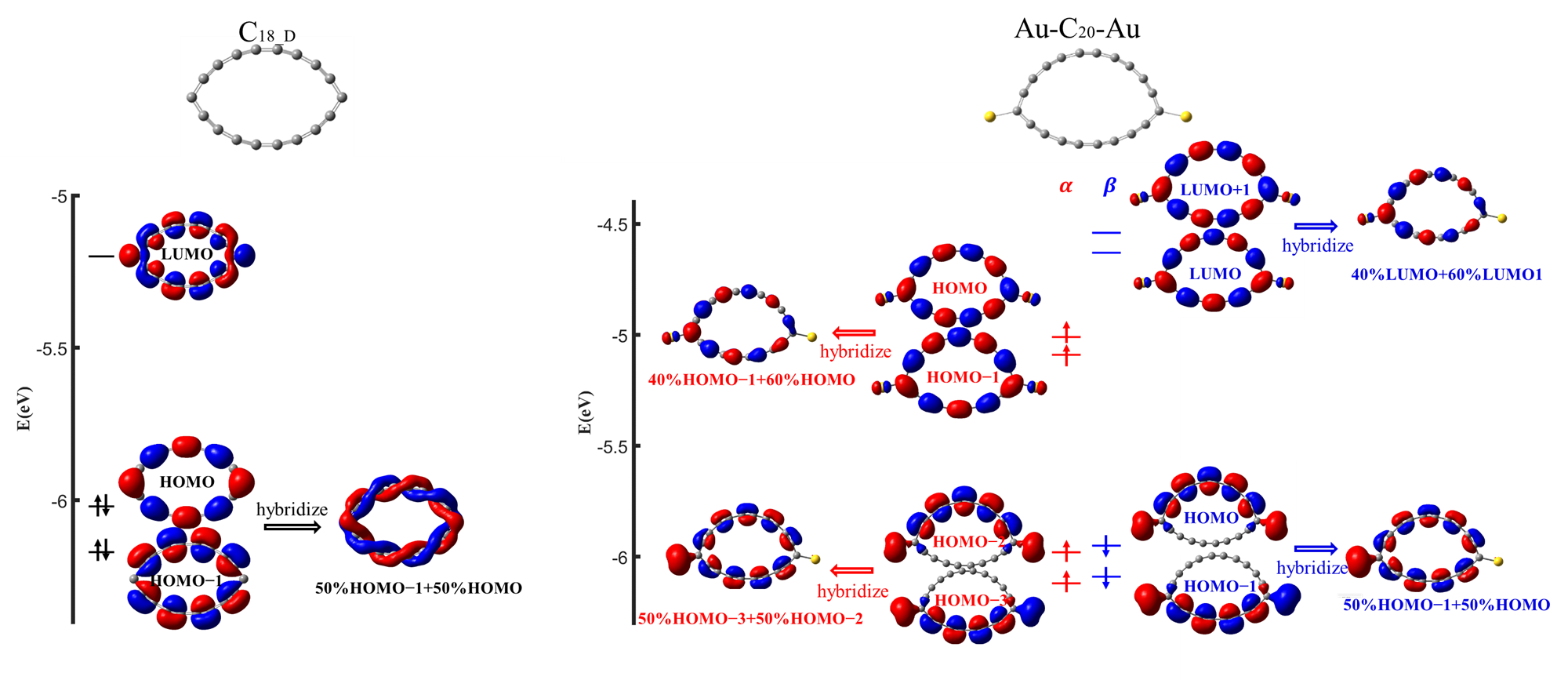


(**a**) (**b**)

**Figure S3** FMOs and the hybrid MOs of the isolated C18_D molecule (**a**) and the Au-C20-Au complex (**b**).


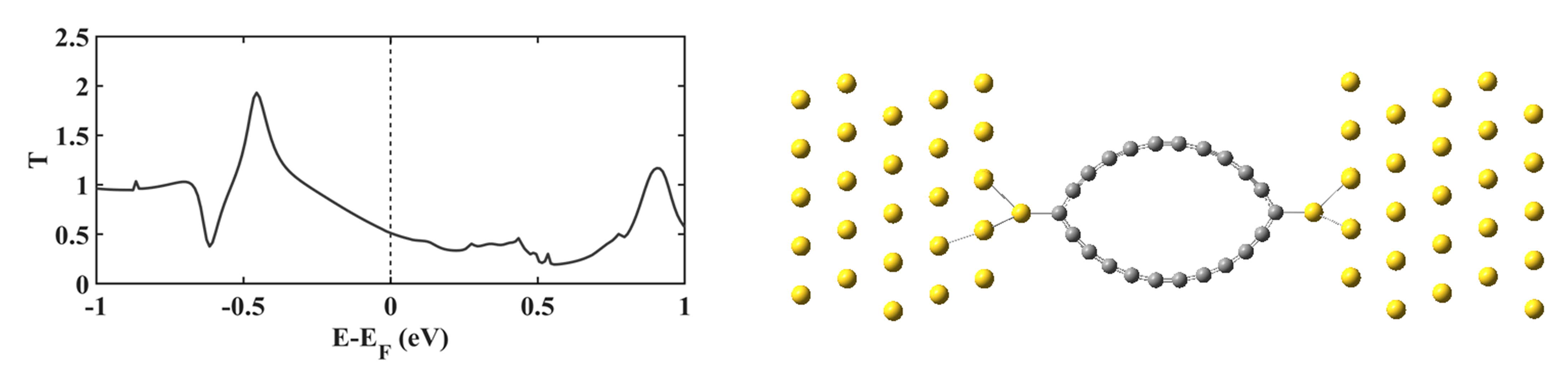


(**a**)


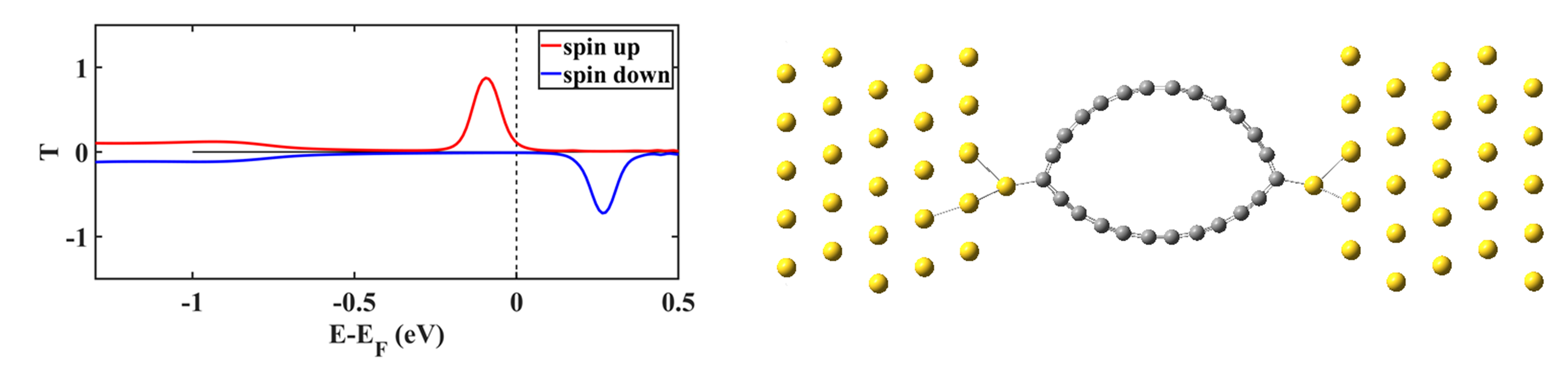


(**b**)


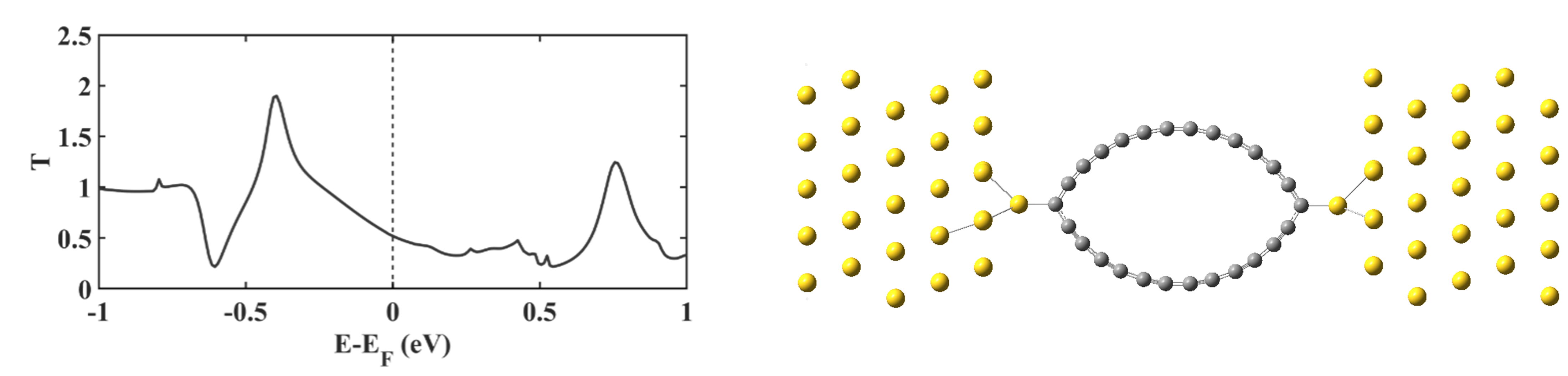


(**c**)


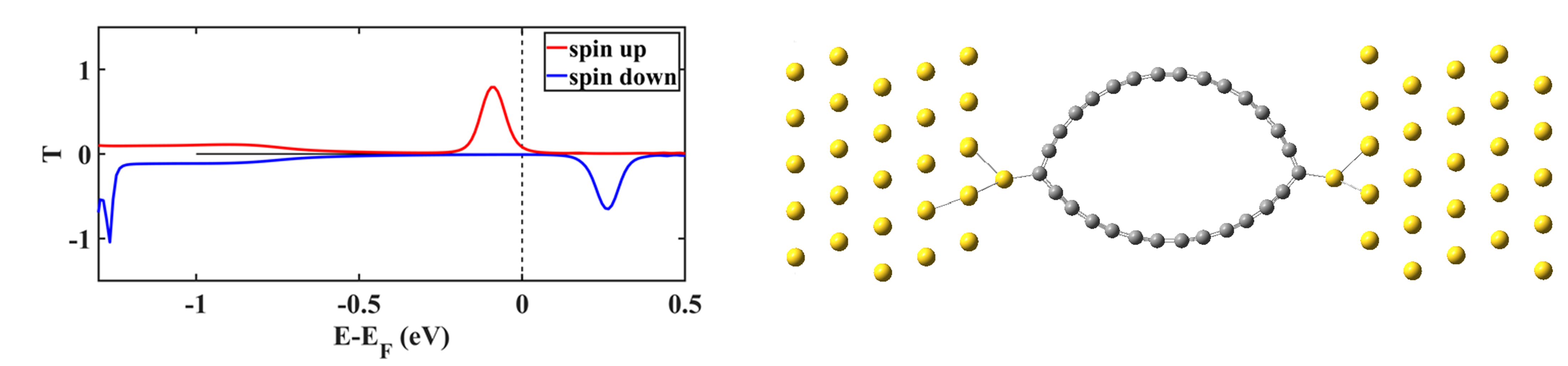


(**d**)

**Figure S4.** The equilibrium transmission spectra of the Au-C_22_-Au (**a**), Au-C_24_-Au (**b**), Au-C_26_-Au (**c**) and Au-C_28_-Au (**d**) molecular junctions are displayed in the left panels, and their corresponding atomic structures are shown in the right panels


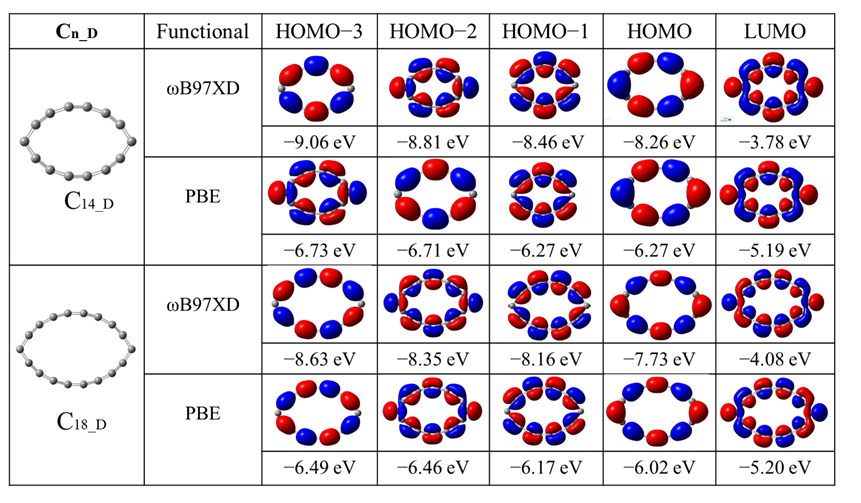


(**a**)


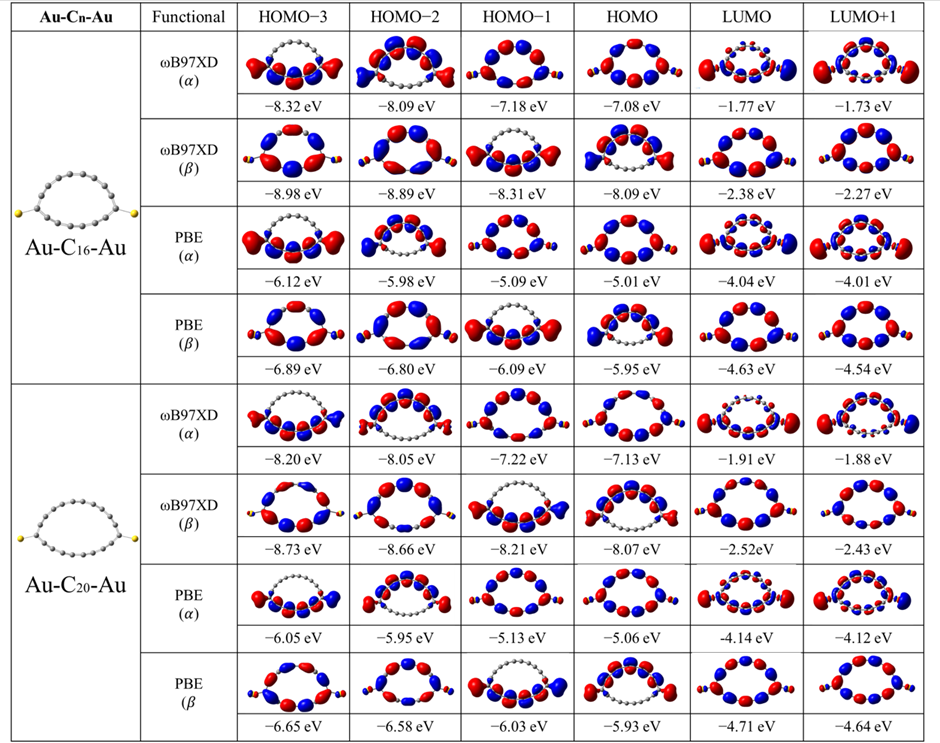


(**b**)

**Figure S5.** FMOs of the isolated C_14_D/18_D_ molecules (**a**) and the Au-C_16/20_-Au complexes (**b**) calculated at both the ωB97XD and PBE levels.

**Cartesian coordinates of the Au-C_n_-Au molecular junctions (unit: Å)**

Au-C_14_-Au

Au 1.48116491 0.85513383 0.00000000

Au 4.44346497 0.85513383 0.00000000

Au 7.40576503 0.85513383 0.00000000

Au 10.36806509 0.85513383 0.00000000

Au 0.00001488 3.42056094 0.00000000

Au -1.48113516 5.98598805 0.00000000

Au -2.96228519 8.55141515 0.00000000

Au -4.44343522 11.11684226 0.00000000

Au 2.96231494 3.42056094 0.00000000

Au 1.48116491 5.98598805 0.00000000

Au 0.00001488 8.55141515 0.00000000

Au -1.48113516 11.11684226 0.00000000

Au 5.92461500 3.42056094 0.00000000

Au 4.44346497 5.98598805 0.00000000

Au 2.96231494 8.55141515 0.00000000

Au 1.48116491 11.11684226 0.00000000

Au 8.88691506 3.42056094 0.00000000

Au 7.40576503 5.98598805 0.00000000

Au 5.92461500 8.55141515 0.00000000

Au 4.44346497 11.11684226 0.00000000

Au -0.00001483 1.71029335 2.41881000

Au 2.96228523 1.71029335 2.41881000

Au 5.92458529 1.71029335 2.41881000

Au 8.88688535 1.71029335 2.41881000

Au -1.48116486 4.27572046 2.41881000

Au -2.96231489 6.84114757 2.41881000

Au -4.44346493 9.40657467 2.41881000

Au -5.92461496 11.97200178 2.41881000

Au 1.48113520 4.27572046 2.41881000

Au -0.00001483 6.84114757 2.41881000

Au -1.48116486 9.40657467 2.41881000

Au -2.96231489 11.97200178 2.41881000

Au 4.44343526 4.27572046 2.41881000

Au 2.96228523 6.84114757 2.41881000

Au 1.48113520 9.40657467 2.41881000

Au -0.00001483 11.97200178 2.41881000

Au 7.40573532 4.27572046 2.41881000

Au 5.92458529 6.84114757 2.41881000

Au 4.44343526 9.40657467 2.41881000

Au 2.96228523 11.97200178 2.41881000

Au 0.00000000 0.00000000 4.83762000

Au 2.96230006 0.00000000 4.83762000

Au 5.92460012 0.00000000 4.83762000

Au 8.88690019 0.00000000 4.83762000

Au -1.48115003 2.56542711 4.83762000

Au -2.96230006 5.13085422 4.83762000

Au -4.44345009 7.69628132 4.83762000

Au -5.92460012 10.26170843 4.83762000

Au 1.48115003 2.56542711 4.83762000

Au 0.00000000 5.13085422 4.83762000

Au -1.48115003 7.69628132 4.83762000

Au -2.96230006 10.26170843 4.83762000

Au 4.44345009 2.56542711 4.83762000

Au 2.96230006 5.13085422 4.83762000

Au 1.48115003 7.69628132 4.83762000

Au 0.00000000 10.26170843 4.83762000

Au 7.40575016 2.56542711 4.83762000

Au 5.92460012 5.13085422 4.83762000

Au 4.44345009 7.69628132 4.83762000

Au 2.96230006 10.26170843 4.83762000

Au 1.48116491 0.85513383 7.25643000

Au 4.44346497 0.85513383 7.25643000

Au 7.40576503 0.85513383 7.25643000

Au 10.36806509 0.85513383 7.25643000

Au 0.00001488 3.42056094 7.25643000

Au -1.48113516 5.98598805 7.25643000

Au -2.96228519 8.55141515 7.25643000

Au -4.44343522 11.11684226 7.25643000

Au 2.96231494 3.42056094 7.25643000

Au 1.48116491 5.98598805 7.25643000

Au 0.00001488 8.55141515 7.25643000

Au -1.48113516 11.11684226 7.25643000

Au 5.92461500 3.42056094 7.25643000

Au 4.44346497 5.98598805 7.25643000

Au 2.96231494 8.55141515 7.25643000

Au 1.48116491 11.11684226 7.25643000

Au 8.88691506 3.42056094 7.25643000

Au 7.40576503 5.98598805 7.25643000

Au 5.92461500 8.55141515 7.25643000

Au 4.44346497 11.11684226 7.25643000

Au 0.00274052 1.69773468 9.62593909

Au 2.98578414 1.67007673 9.62979237

Au 5.92543115 1.70440831 9.63633913

Au 8.87261743 1.66556790 9.63010255

Au -1.58322102 4.21076864 9.61983894

Au -2.96112312 6.84513460 9.61897402

Au -4.44430716 9.40700337 9.63221622

Au -5.92424354 11.96472145 9.63487826

Au 1.59283436 4.20788802 9.61592552

Au 0.00554474 6.97093109 9.62560513

Au -1.49909859 9.44417971 9.63126788

Au -2.96536211 11.97005051 9.63737250

Au 4.48113757 4.27721658 9.62656508

Au 2.97664571 6.84359983 9.62373882

Au 1.50754487 9.44562808 9.63198998

Au -0.00471672 11.97147696 9.63849355

Au 7.37612071 4.26997277 9.63106347

Au 5.93403713 6.84533227 9.63524380

Au 4.45665374 9.40373984 9.63232868

Au 2.97507324 11.96686519 9.63645618

Au 0.00920090 5.12506599 11.58540887

C 0.00919290 7.45313699 17.79679287

C 0.00920790 7.03034999 19.04852287

C 0.00904990 6.25824499 20.01622287

C 0.00913190 5.12500299 20.77306687

C 0.00905490 3.99162599 20.01636687

C 0.00920990 3.21993299 19.04835687

C 0.00918890 2.79654399 17.79681087

C 0.00916190 2.79685899 16.55724487

C 0.00914590 3.21945199 15.30542987

C 0.00923190 5.12517999 13.58126787

C 0.00928890 6.25856899 14.33787487

C 0.00929390 3.99192399 14.33801687

C 0.00914390 7.03063699 15.30559187

C 0.00916490 7.45382599 16.55722687

Au 0.00922090 5.12509899 22.77035587

Au 0.00274052 1.69773468 24.72982565

Au 2.98578414 1.67007673 24.72597237

Au 5.92543115 1.70440831 24.71942561

Au 8.87261743 1.66556790 24.72566219

Au -1.58322102 4.21076864 24.73592580

Au -2.96112312 6.84513460 24.73679072

Au -4.44430716 9.40700337 24.72354852

Au -5.92424354 11.96472145 24.72088648

Au 1.59283436 4.20788802 24.73983922

Au 0.00554474 6.97093109 24.73015961

Au -1.49909859 9.44417971 24.72449686

Au -2.96536211 11.97005051 24.71839224

Au 4.48113757 4.27721658 24.72919966

Au 2.97664571 6.84359983 24.73202592

Au 1.50754487 9.44562808 24.72377476

Au -0.00471672 11.97147696 24.71727119

Au 7.37612071 4.26997277 24.72470127

Au 5.93403713 6.84533227 24.72052094

Au 4.45665374 9.40373984 24.72343606

Au 2.97507324 11.96686519 24.71930856

Au 0.00000000 0.00000000 27.09933474

Au 2.96230006 0.00000000 27.09933474

Au 5.92460012 0.00000000 27.09933474

Au 8.88690019 0.00000000 27.09933474

Au -1.48115003 2.56542711 27.09933474

Au -2.96230006 5.13085422 27.09933474

Au -4.44345009 7.69628132 27.09933474

Au -5.92460012 10.26170843 27.09933474

Au 1.48115003 2.56542711 27.09933474

Au 0.00000000 5.13085422 27.09933474

Au -1.48115003 7.69628132 27.09933474

Au -2.96230006 10.26170843 27.09933474

Au 4.44345009 2.56542711 27.09933474

Au 2.96230006 5.13085422 27.09933474

Au 1.48115003 7.69628132 27.09933474

Au 0.00000000 10.26170843 27.09933474

Au 7.40575016 2.56542711 27.09933474

Au 5.92460012 5.13085422 27.09933474

Au 4.44345009 7.69628132 27.09933474

Au 2.96230006 10.26170843 27.09933474

Au 1.48116491 0.85513383 29.51814474

Au 4.44346497 0.85513383 29.51814474

Au 7.40576503 0.85513383 29.51814474

Au 10.36806509 0.85513383 29.51814474

Au 0.00001488 3.42056094 29.51814474

Au -1.48113516 5.98598805 29.51814474

Au -2.96228519 8.55141515 29.51814474

Au -4.44343522 11.11684226 29.51814474

Au 2.96231494 3.42056094 29.51814474

Au 1.48116491 5.98598805 29.51814474

Au 0.00001488 8.55141515 29.51814474

Au -1.48113516 11.11684226 29.51814474

Au 5.92461500 3.42056094 29.51814474

Au 4.44346497 5.98598805 29.51814474

Au 2.96231494 8.55141515 29.51814474

Au 1.48116491 11.11684226 29.51814474

Au 8.88691506 3.42056094 29.51814474

Au 7.40576503 5.98598805 29.51814474

Au 5.92461500 8.55141515 29.51814474

Au 4.44346497 11.11684226 29.51814474

Au -0.00001483 1.71029335 31.93695474

Au 2.96228523 1.71029335 31.93695474

Au 5.92458529 1.71029335 31.93695474

Au 8.88688535 1.71029335 31.93695474

Au -1.48116486 4.27572046 31.93695474

Au -2.96231489 6.84114757 31.93695474

Au -4.44346493 9.40657467 31.93695474

Au -5.92461496 11.97200178 31.93695474

Au 1.48113520 4.27572046 31.93695474

Au -0.00001483 6.84114757 31.93695474

Au -1.48116486 9.40657467 31.93695474

Au -2.96231489 11.97200178 31.93695474

Au 4.44343526 4.27572046 31.93695474

Au 2.96228523 6.84114757 31.93695474

Au 1.48113520 9.40657467 31.93695474

Au -0.00001483 11.97200178 31.93695474

Au 7.40573532 4.27572046 31.93695474

Au 5.92458529 6.84114757 31.93695474

Au 4.44343526 9.40657467 31.93695474

Au 2.96228523 11.97200178 31.93695474

Au 0.00000000 0.00000000 34.35576474

Au 2.96230006 0.00000000 34.35576474

Au 5.92460012 0.00000000 34.35576474

Au 8.88690019 0.00000000 34.35576474

Au -1.48115003 2.56542711 34.35576474

Au -2.96230006 5.13085422 34.35576474

Au -4.44345009 7.69628132 34.35576474

Au -5.92460012 10.26170843 34.35576474

Au 1.48115003 2.56542711 34.35576474

Au 0.00000000 5.13085422 34.35576474

Au -1.48115003 7.69628132 34.35576474

Au -2.96230006 10.26170843 34.35576474

Au 4.44345009 2.56542711 34.35576474

Au 2.96230006 5.13085422 34.35576474

Au 1.48115003 7.69628132 34.35576474

Au 0.00000000 10.26170843 34.35576474

Au 7.40575016 2.56542711 34.35576474

Au 5.92460012 5.13085422 34.35576474

Au 4.44345009 7.69628132 34.35576474

Au 2.96230006 10.26170843 34.35576474

Tv 11.84920025 0.00000000 0.00000000

Tv -7.40575016 12.82713554 0.00000000

Tv 0.00000000 0.00000000 36.77457474

Au-C_16_-Au

Au 1.48116553 0.85513403 0.00000000

Au 4.44346696 0.85513403 0.00000000

Au 7.40576839 0.85513403 0.00000000

Au 10.36806983 0.85513403 0.00000000

Au 0.00001482 3.42056258 0.00000000

Au -1.48113590 5.98599113 0.00000000

Au -2.96228661 8.55141915 0.00000000

Au -4.44343733 11.11684769 0.00000000

Au 2.96231625 3.42056258 0.00000000

Au 1.48116553 5.98599113 0.00000000

Au 0.00001482 8.55141915 0.00000000

Au -1.48113590 11.11684769 0.00000000

Au 5.92461768 3.42056258 0.00000000

Au 4.44346696 5.98599113 0.00000000

Au 2.96231625 8.55141915 0.00000000

Au 1.48116553 11.11684769 0.00000000

Au 8.88691911 3.42056258 0.00000000

Au 7.40576839 5.98599113 0.00000000

Au 5.92461768 8.55141915 0.00000000

Au 4.44346696 11.11684769 0.00000000

Au -0.00001482 1.71029399 2.41881100

Au 2.96228661 1.71029399 2.41881100

Au 5.92458804 1.71029399 2.41881100

Au 8.88688948 1.71029399 2.41881100

Au -1.48116553 4.27572254 2.41881100

Au -2.96231625 6.84115056 2.41881100

Au -4.44346696 9.40657911 2.41881100

Au -5.92461768 11.97200765 2.41881100

Au 1.48113590 4.27572254 2.41881100

Au -0.00001482 6.84115056 2.41881100

Au -1.48116553 9.40657911 2.41881100

Au -2.96231625 11.97200765 2.41881100

Au 4.44343733 4.27572254 2.41881100

Au 2.96228661 6.84115056 2.41881100

Au 1.48113590 9.40657911 2.41881100

Au -0.00001482 11.97200765 2.41881100

Au 7.40573876 4.27572254 2.41881100

Au 5.92458804 6.84115056 2.41881100

Au 4.44343733 9.40657911 2.41881100

Au 2.96228661 11.97200765 2.41881100

Au 0.00000000 0.00000000 4.83762252

Au 2.96230143 0.00000000 4.83762252

Au 5.92460286 0.00000000 4.83762252

Au 8.88690429 0.00000000 4.83762252

Au -1.48115072 2.56542855 4.83762252

Au -2.96230143 5.13085657 4.83762252

Au -4.44345215 7.69628512 4.83762252

Au -5.92460286 10.26171314 4.83762252

Au 1.48115072 2.56542855 4.83762252

Au 0.00000000 5.13085657 4.83762252

Au -1.48115072 7.69628512 4.83762252

Au -2.96230143 10.26171314 4.83762252

Au 4.44345215 2.56542855 4.83762252

Au 2.96230143 5.13085657 4.83762252

Au 1.48115072 7.69628512 4.83762252

Au 0.00000000 10.26171314 4.83762252

Au 7.40575358 2.56542855 4.83762252

Au 5.92460286 5.13085657 4.83762252

Au 4.44345215 7.69628512 4.83762252

Au 2.96230143 10.26171314 4.83762252

Au 1.48116553 0.85513403 7.25643352

Au 4.44346696 0.85513403 7.25643352

Au 7.40576839 0.85513403 7.25643352

Au 10.36806983 0.85513403 7.25643352

Au 0.00001482 3.42056258 7.25643352

Au -1.48113590 5.98599113 7.25643352

Au -2.96228661 8.55141915 7.25643352

Au -4.44343733 11.11684769 7.25643352

Au 2.96231625 3.42056258 7.25643352

Au 1.48116553 5.98599113 7.25643352

Au 0.00001482 8.55141915 7.25643352

Au -1.48113590 11.11684769 7.25643352

Au 5.92461768 3.42056258 7.25643352

Au 4.44346696 5.98599113 7.25643352

Au 2.96231625 8.55141915 7.25643352

Au 1.48116553 11.11684769 7.25643352

Au 8.88691911 3.42056258 7.25643352

Au 7.40576839 5.98599113 7.25643352

Au 5.92461768 8.55141915 7.25643352

Au 4.44346696 11.11684769 7.25643352

Au 0.00274061 1.69773555 9.62594371

Au 2.98578579 1.67007758 9.62979665

Au 5.92543420 1.70440901 9.63634363

Au 8.87262180 1.66556846 9.63010728

Au -1.58322160 4.21077079 9.61984336

Au -2.96112454 6.84513791 9.61897868

Au -4.44430941 9.40700774 9.63222081

Au -5.92424620 11.96472723 9.63488258

Au 1.59283516 4.20788995 9.61593009

Au 0.00554472 6.97093445 9.62560980

Au -1.49909935 9.44418403 9.63127253

Au -2.96536325 11.97005605 9.63737712

Au 4.48113962 4.27721852 9.62656973

Au 2.97664690 6.84360329 9.62374339

Au 1.50754555 9.44563239 9.63199433

Au -0.00471656 11.97148271 9.63849791

Au 7.37612441 4.26997461 9.63106827

Au 5.93403968 6.84533529 9.63524824

Au 4.45665565 9.40374430 9.63233300

Au 2.97507471 11.96687093 9.63646058

Au 0.00920080 5.12506843 11.58540610

C 0.00831179 5.63864183 21.60956205

C 0.00940983 4.15766098 19.48507785

C 0.01002791 3.84736890 18.16957443

C 0.01039516 3.84716676 16.94943986

C 0.01036605 4.15762817 15.63397825

C 0.00914577 8.75627530 15.72900525

C 0.00906269 9.15536808 16.88417908

C 0.00851499 8.75631711 19.38994501

C 0.00823082 7.95389495 20.47083029

C 0.00815303 6.93793602 21.16234007

C 0.00885366 4.72418703 20.57057861

C 0.00872402 9.15514107 18.23467964

C 0.00954795 7.95406270 14.64798451

C 0.00978872 6.93800904 13.95662131

C 0.00962309 5.63870850 13.50945436

C 0.01018613 4.72438811 14.54858173

Au 0.00853298 5.12510864 23.53362883

Au 0.00274061 1.69773555 25.52253940

Au 2.98578579 1.67007758 25.51868646

Au 5.92543420 1.70440901 25.51213948

Au 8.87262180 1.66556846 25.51837636

Au -1.58322160 4.21077079 25.52863976

Au -2.96112454 6.84513791 25.52950496

Au -4.44430941 9.40700774 25.51626230

Au -5.92424620 11.96472723 25.51360054

Au 1.59283516 4.20788995 25.53255302

Au 0.00554472 6.97093445 25.52287384

Au -1.49909935 9.44418403 25.51721111

Au -2.96536325 11.97005605 25.51110600

Au 4.48113962 4.27721852 25.52191391

Au 2.97664690 6.84360329 25.52473972

Au 1.50754555 9.44563239 25.51648879

Au -0.00471656 11.97148271 25.50998520

Au 7.37612441 4.26997461 25.51741538

Au 5.93403968 6.84533529 25.51323488

Au 4.45665565 9.40374430 25.51615011

Au 2.97507471 11.96687093 25.51202253

Au 0.00000000 0.00000000 27.89205013

Au 2.96230143 0.00000000 27.89205013

Au 5.92460286 0.00000000 27.89205013

Au 8.88690429 0.00000000 27.89205013

Au -1.48115072 2.56542855 27.89205013

Au -2.96230143 5.13085657 27.89205013

Au -4.44345215 7.69628512 27.89205013

Au -5.92460286 10.26171314 27.89205013

Au 1.48115072 2.56542855 27.89205013

Au 0.00000000 5.13085657 27.89205013

Au -1.48115072 7.69628512 27.89205013

Au -2.96230143 10.26171314 27.89205013

Au 4.44345215 2.56542855 27.89205013

Au 2.96230143 5.13085657 27.89205013

Au 1.48115072 7.69628512 27.89205013

Au 0.00000000 10.26171314 27.89205013

Au 7.40575358 2.56542855 27.89205013

Au 5.92460286 5.13085657 27.89205013

Au 4.44345215 7.69628512 27.89205013

Au 2.96230143 10.26171314 27.89205013

Au 1.48116553 0.85513403 30.31086112

Au 4.44346696 0.85513403 30.31086112

Au 7.40576839 0.85513403 30.31086112

Au 10.36806983 0.85513403 30.31086112

Au 0.00001482 3.42056258 30.31086112

Au -1.48113590 5.98599113 30.31086112

Au -2.96228661 8.55141915 30.31086112

Au -4.44343733 11.11684769 30.31086112

Au 2.96231625 3.42056258 30.31086112

Au 1.48116553 5.98599113 30.31086112

Au 0.00001482 8.55141915 30.31086112

Au -1.48113590 11.11684769 30.31086112

Au 5.92461768 3.42056258 30.31086112

Au 4.44346696 5.98599113 30.31086112

Au 2.96231625 8.55141915 30.31086112

Au 1.48116553 11.11684769 30.31086112

Au 8.88691911 3.42056258 30.31086112

Au 7.40576839 5.98599113 30.31086112

Au 5.92461768 8.55141915 30.31086112

Au 4.44346696 11.11684769 30.31086112

Au -0.00001482 1.71029399 32.72967212

Au 2.96228661 1.71029399 32.72967212

Au 5.92458804 1.71029399 32.72967212

Au 8.88688948 1.71029399 32.72967212

Au -1.48116553 4.27572254 32.72967212

Au -2.96231625 6.84115056 32.72967212

Au -4.44346696 9.40657911 32.72967212

Au -5.92461768 11.97200765 32.72967212

Au 1.48113590 4.27572254 32.72967212

Au -0.00001482 6.84115056 32.72967212

Au -1.48116553 9.40657911 32.72967212

Au -2.96231625 11.97200765 32.72967212

Au 4.44343733 4.27572254 32.72967212

Au 2.96228661 6.84115056 32.72967212

Au 1.48113590 9.40657911 32.72967212

Au -0.00001482 11.97200765 32.72967212

Au 7.40573876 4.27572254 32.72967212

Au 5.92458804 6.84115056 32.72967212

Au 4.44343733 9.40657911 32.72967212

Au 2.96228661 11.97200765 32.72967212

Au 0.00000000 0.00000000 35.14848311

Au 2.96230143 0.00000000 35.14848311

Au 5.92460286 0.00000000 35.14848311

Au 8.88690429 0.00000000 35.14848311

Au -1.48115072 2.56542855 35.14848311

Au -2.96230143 5.13085657 35.14848311

Au -4.44345215 7.69628512 35.14848311

Au -5.92460286 10.26171314 35.14848311

Au 1.48115072 2.56542855 35.14848311

Au 0.00000000 5.13085657 35.14848311

Au -1.48115072 7.69628512 35.14848311

Au -2.96230143 10.26171314 35.14848311

Au 4.44345215 2.56542855 35.14848311

Au 2.96230143 5.13085657 35.14848311

Au 1.48115072 7.69628512 35.14848311

Au 0.00000000 10.26171314 35.14848311

Au 7.40575358 2.56542855 35.14848311

Au 5.92460286 5.13085657 35.14848311

Au 4.44345215 7.69628512 35.14848311

Au 2.96230143 10.26171314 35.14848311

Tv 11.84920025 0.00000000 0.00000000

Tv -7.40575016 12.82713554 0.00000000

Tv 0.00000000 0.00000000 37.56729411

Au-C_18_-Au

Au 1.48116553 0.85513403 0.00000000

Au 4.44346696 0.85513403 0.00000000

Au 7.40576839 0.85513403 0.00000000

Au 10.36806983 0.85513403 0.00000000

Au 0.00001482 3.42056258 0.00000000

Au -1.48113590 5.98599113 0.00000000

Au -2.96228661 8.55141915 0.00000000

Au -4.44343733 11.11684769 0.00000000

Au 2.96231625 3.42056258 0.00000000

Au 1.48116553 5.98599113 0.00000000

Au 0.00001482 8.55141915 0.00000000

Au -1.48113590 11.11684769 0.00000000

Au 5.92461768 3.42056258 0.00000000

Au 4.44346696 5.98599113 0.00000000

Au 2.96231625 8.55141915 0.00000000

Au 1.48116553 11.11684769 0.00000000

Au 8.88691911 3.42056258 0.00000000

Au 7.40576839 5.98599113 0.00000000

Au 5.92461768 8.55141915 0.00000000

Au 4.44346696 11.11684769 0.00000000

Au -0.00001482 1.71029399 2.41881100

Au 2.96228661 1.71029399 2.41881100

Au 5.92458804 1.71029399 2.41881100

Au 8.88688948 1.71029399 2.41881100

Au -1.48116553 4.27572254 2.41881100

Au -2.96231625 6.84115056 2.41881100

Au -4.44346696 9.40657911 2.41881100

Au -5.92461768 11.97200765 2.41881100

Au 1.48113590 4.27572254 2.41881100

Au -0.00001482 6.84115056 2.41881100

Au -1.48116553 9.40657911 2.41881100

Au -2.96231625 11.97200765 2.41881100

Au 4.44343733 4.27572254 2.41881100

Au 2.96228661 6.84115056 2.41881100

Au 1.48113590 9.40657911 2.41881100

Au -0.00001482 11.97200765 2.41881100

Au 7.40573876 4.27572254 2.41881100

Au 5.92458804 6.84115056 2.41881100

Au 4.44343733 9.40657911 2.41881100

Au 2.96228661 11.97200765 2.41881100

Au 0.00000000 0.00000000 4.83762252

Au 2.96230143 0.00000000 4.83762252

Au 5.92460286 0.00000000 4.83762252

Au 8.88690429 0.00000000 4.83762252

Au -1.48115072 2.56542855 4.83762252

Au -2.96230143 5.13085657 4.83762252

Au -4.44345215 7.69628512 4.83762252

Au -5.92460286 10.26171314 4.83762252

Au 1.48115072 2.56542855 4.83762252

Au 0.00000000 5.13085657 4.83762252

Au -1.48115072 7.69628512 4.83762252

Au -2.96230143 10.26171314 4.83762252

Au 4.44345215 2.56542855 4.83762252

Au 2.96230143 5.13085657 4.83762252

Au 1.48115072 7.69628512 4.83762252

Au 0.00000000 10.26171314 4.83762252

Au 7.40575358 2.56542855 4.83762252

Au 5.92460286 5.13085657 4.83762252

Au 4.44345215 7.69628512 4.83762252

Au 2.96230143 10.26171314 4.83762252

Au 1.48116553 0.85513403 7.25643352

Au 4.44346696 0.85513403 7.25643352

Au 7.40576839 0.85513403 7.25643352

Au 10.36806983 0.85513403 7.25643352

Au 0.00001482 3.42056258 7.25643352

Au -1.48113590 5.98599113 7.25643352

Au -2.96228661 8.55141915 7.25643352

Au -4.44343733 11.11684769 7.25643352

Au 2.96231625 3.42056258 7.25643352

Au 1.48116553 5.98599113 7.25643352

Au 0.00001482 8.55141915 7.25643352

Au -1.48113590 11.11684769 7.25643352

Au 5.92461768 3.42056258 7.25643352

Au 4.44346696 5.98599113 7.25643352

Au 2.96231625 8.55141915 7.25643352

Au 1.48116553 11.11684769 7.25643352

Au 8.88691911 3.42056258 7.25643352

Au 7.40576839 5.98599113 7.25643352

Au 5.92461768 8.55141915 7.25643352

Au 4.44346696 11.11684769 7.25643352

Au 0.00274061 1.69773555 9.62594371

Au 2.98578579 1.67007758 9.62979665

Au 5.92543420 1.70440901 9.63634363

Au 8.87262180 1.66556846 9.63010728

Au -1.58322160 4.21077079 9.61984336

Au -2.96112454 6.84513791 9.61897868

Au -4.44430941 9.40700774 9.63222081

Au -5.92424620 11.96472723 9.63488258

Au 1.59283516 4.20788995 9.61593009

Au 0.00554472 6.97093445 9.62560980

Au -1.49909935 9.44418403 9.63127253

Au -2.96536325 11.97005605 9.63737712

Au 4.48113962 4.27721852 9.62656973

Au 2.97664690 6.84360329 9.62374339

Au 1.50754555 9.44563239 9.63199433

Au -0.00471656 11.97148271 9.63849791

Au 7.37612441 4.26997461 9.63106827

Au 5.93403968 6.84533529 9.63524824

Au 4.45665565 9.40374430 9.63233300

Au 2.97507471 11.96687093 9.63646058

Au 0.00920080 5.12506843 11.58541457

C 0.00865893 2.20359514 18.88990955

C 0.01073912 2.20363906 17.58011701

C 0.01083014 2.50937809 16.37302349

C 0.01337601 3.13135927 15.21868418

C 0.01216314 3.99017691 14.31985386

C 0.01126301 5.12404659 13.58563736

C 0.01150802 6.27873304 14.32426879

C 0.00957705 7.13460315 15.20944527

C 0.00998505 7.76816062 16.37723733

C 0.01000515 8.07378376 17.57127129

C 0.00941988 8.07375095 18.89877697

C 0.00941777 7.76822253 20.09283421

C 0.00983793 7.13463438 21.26061198

C 0.00801280 6.27879601 22.14581810

C 0.00818214 5.12408363 22.88441883

C 0.00737091 3.99024782 22.15013830

C 0.00614216 3.13141537 21.25132281

C 0.00852081 2.50947335 20.09696656

Au 0.01005701 5.12504938 24.88464957

Au 0.00274061 1.69773555 26.84412042

Au 2.98578579 1.67007758 26.84026695

Au 5.92543420 1.70440901 26.83372050

Au 8.87262180 1.66556846 26.83995685

Au -1.58322160 4.21077079 26.85022025

Au -2.96112454 6.84513791 26.85108545

Au -4.44430941 9.40700774 26.83784332

Au -5.92424620 11.96472723 26.83518103

Au 1.59283516 4.20788995 26.85413404

Au 0.00554472 6.97093445 26.84445433

Au -1.49909935 9.44418403 26.83879161

Au -2.96536325 11.97005605 26.83268702

Au 4.48113962 4.27721852 26.84349440

Au 2.97664690 6.84360329 26.84632074

Au 1.50754555 9.44563239 26.83806928

Au -0.00471656 11.97148271 26.83156569

Au 7.37612441 4.26997461 26.83899587

Au 5.93403968 6.84533529 26.83481537

Au 4.45665565 9.40374430 26.83773061

Au 2.97507471 11.96687093 26.83360302

Au 0.00000000 0.00000000 29.21363062

Au 2.96230143 0.00000000 29.21363062

Au 5.92460286 0.00000000 29.21363062

Au 8.88690429 0.00000000 29.21363062

Au -1.48115072 2.56542855 29.21363062

Au -2.96230143 5.13085657 29.21363062

Au -4.44345215 7.69628512 29.21363062

Au -5.92460286 10.26171314 29.21363062

Au 1.48115072 2.56542855 29.21363062

Au 0.00000000 5.13085657 29.21363062

Au -1.48115072 7.69628512 29.21363062

Au -2.96230143 10.26171314 29.21363062

Au 4.44345215 2.56542855 29.21363062

Au 2.96230143 5.13085657 29.21363062

Au 1.48115072 7.69628512 29.21363062

Au 0.00000000 10.26171314 29.21363062

Au 7.40575358 2.56542855 29.21363062

Au 5.92460286 5.13085657 29.21363062

Au 4.44345215 7.69628512 29.21363062

Au 2.96230143 10.26171314 29.21363062

Au 1.48116553 0.85513403 31.63244161

Au 4.44346696 0.85513403 31.63244161

Au 7.40576839 0.85513403 31.63244161

Au 10.36806983 0.85513403 31.63244161

Au 0.00001482 3.42056258 31.63244161

Au -1.48113590 5.98599113 31.63244161

Au -2.96228661 8.55141915 31.63244161

Au -4.44343733 11.11684769 31.63244161

Au 2.96231625 3.42056258 31.63244161

Au 1.48116553 5.98599113 31.63244161

Au 0.00001482 8.55141915 31.63244161

Au -1.48113590 11.11684769 31.63244161

Au 5.92461768 3.42056258 31.63244161

Au 4.44346696 5.98599113 31.63244161

Au 2.96231625 8.55141915 31.63244161

Au 1.48116553 11.11684769 31.63244161

Au 8.88691911 3.42056258 31.63244161

Au 7.40576839 5.98599113 31.63244161

Au 5.92461768 8.55141915 31.63244161

Au 4.44346696 11.11684769 31.63244161

Au -0.00001482 1.71029399 34.05125261

Au 2.96228661 1.71029399 34.05125261

Au 5.92458804 1.71029399 34.05125261

Au 8.88688948 1.71029399 34.05125261

Au -1.48116553 4.27572254 34.05125261

Au -2.96231625 6.84115056 34.05125261

Au -4.44346696 9.40657911 34.05125261

Au -5.92461768 11.97200765 34.05125261

Au 1.48113590 4.27572254 34.05125261

Au -0.00001482 6.84115056 34.05125261

Au -1.48116553 9.40657911 34.05125261

Au -2.96231625 11.97200765 34.05125261

Au 4.44343733 4.27572254 34.05125261

Au 2.96228661 6.84115056 34.05125261

Au 1.48113590 9.40657911 34.05125261

Au -0.00001482 11.97200765 34.05125261

Au 7.40573876 4.27572254 34.05125261

Au 5.92458804 6.84115056 34.05125261

Au 4.44343733 9.40657911 34.05125261

Au 2.96228661 11.97200765 34.05125261

Au 0.00000000 0.00000000 36.47006413

Au 2.96230143 0.00000000 36.47006413

Au 5.92460286 0.00000000 36.47006413

Au 8.88690429 0.00000000 36.47006413

Au -1.48115072 2.56542855 36.47006413

Au -2.96230143 5.13085657 36.47006413

Au -4.44345215 7.69628512 36.47006413

Au -5.92460286 10.26171314 36.47006413

Au 1.48115072 2.56542855 36.47006413

Au 0.00000000 5.13085657 36.47006413

Au -1.48115072 7.69628512 36.47006413

Au -2.96230143 10.26171314 36.47006413

Au 4.44345215 2.56542855 36.47006413

Au 2.96230143 5.13085657 36.47006413

Au 1.48115072 7.69628512 36.47006413

Au 0.00000000 10.26171314 36.47006413

Au 7.40575358 2.56542855 36.47006413

Au 5.92460286 5.13085657 36.47006413

Au 4.44345215 7.69628512 36.47006413

Au 2.96230143 10.26171314 36.47006413

Tv 11.84920025 0.00000000 0.00000000

Tv -7.40575016 12.82713554 0.00000000

Tv 0.00000000 0.00000000 38.888875

Au-C_20_-Au

Au 1.48116491 0.85513383 0.00000000

Au 4.44346497 0.85513383 0.00000000

Au 7.40576503 0.85513383 0.00000000

Au 10.36806509 0.85513383 0.00000000

Au 0.00001488 3.42056094 0.00000000

Au -1.48113516 5.98598805 0.00000000

Au -2.96228519 8.55141515 0.00000000

Au -4.44343522 11.11684226 0.00000000

Au 2.96231494 3.42056094 0.00000000

Au 1.48116491 5.98598805 0.00000000

Au 0.00001488 8.55141515 0.00000000

Au -1.48113516 11.11684226 0.00000000

Au 5.92461500 3.42056094 0.00000000

Au 4.44346497 5.98598805 0.00000000

Au 2.96231494 8.55141515 0.00000000

Au 1.48116491 11.11684226 0.00000000

Au 8.88691506 3.42056094 0.00000000

Au 7.40576503 5.98598805 0.00000000

Au 5.92461500 8.55141515 0.00000000

Au 4.44346497 11.11684226 0.00000000

Au -0.00001483 1.71029335 2.41881000

Au 2.96228523 1.71029335 2.41881000

Au 5.92458529 1.71029335 2.41881000

Au 8.88688535 1.71029335 2.41881000

Au -1.48116486 4.27572046 2.41881000

Au -2.96231489 6.84114757 2.41881000

Au -4.44346493 9.40657467 2.41881000

Au -5.92461496 11.97200178 2.41881000

Au 1.48113520 4.27572046 2.41881000

Au -0.00001483 6.84114757 2.41881000

Au -1.48116486 9.40657467 2.41881000

Au -2.96231489 11.97200178 2.41881000

Au 4.44343526 4.27572046 2.41881000

Au 2.96228523 6.84114757 2.41881000

Au 1.48113520 9.40657467 2.41881000

Au -0.00001483 11.97200178 2.41881000

Au 7.40573532 4.27572046 2.41881000

Au 5.92458529 6.84114757 2.41881000

Au 4.44343526 9.40657467 2.41881000

Au 2.96228523 11.97200178 2.41881000

Au 0.00000000 0.00000000 4.83762000

Au 2.96230006 0.00000000 4.83762000

Au 5.92460012 0.00000000 4.83762000

Au 8.88690019 0.00000000 4.83762000

Au -1.48115003 2.56542711 4.83762000

Au -2.96230006 5.13085422 4.83762000

Au -4.44345009 7.69628132 4.83762000

Au -5.92460012 10.26170843 4.83762000

Au 1.48115003 2.56542711 4.83762000

Au 0.00000000 5.13085422 4.83762000

Au -1.48115003 7.69628132 4.83762000

Au -2.96230006 10.26170843 4.83762000

Au 4.44345009 2.56542711 4.83762000

Au 2.96230006 5.13085422 4.83762000

Au 1.48115003 7.69628132 4.83762000

Au 0.00000000 10.26170843 4.83762000

Au 7.40575016 2.56542711 4.83762000

Au 5.92460012 5.13085422 4.83762000

Au 4.44345009 7.69628132 4.83762000

Au 2.96230006 10.26170843 4.83762000

Au 1.48116491 0.85513383 7.25643000

Au 4.44346497 0.85513383 7.25643000

Au 7.40576503 0.85513383 7.25643000

Au 10.36806509 0.85513383 7.25643000

Au 0.00001488 3.42056094 7.25643000

Au -1.48113516 5.98598805 7.25643000

Au -2.96228519 8.55141515 7.25643000

Au -4.44343522 11.11684226 7.25643000

Au 2.96231494 3.42056094 7.25643000

Au 1.48116491 5.98598805 7.25643000

Au 0.00001488 8.55141515 7.25643000

Au -1.48113516 11.11684226 7.25643000

Au 5.92461500 3.42056094 7.25643000

Au 4.44346497 5.98598805 7.25643000

Au 2.96231494 8.55141515 7.25643000

Au 1.48116491 11.11684226 7.25643000

Au 8.88691506 3.42056094 7.25643000

Au 7.40576503 5.98598805 7.25643000

Au 5.92461500 8.55141515 7.25643000

Au 4.44346497 11.11684226 7.25643000

Au 0.00274052 1.69773468 9.62593909

Au 2.98578414 1.67007673 9.62979237

Au 5.92543115 1.70440831 9.63633913

Au 8.87261743 1.66556790 9.63010255

Au -1.58322102 4.21076864 9.61983894

Au -2.96112312 6.84513460 9.61897402

Au -4.44430716 9.40700337 9.63221622

Au -5.92424354 11.96472145 9.63487826

Au 1.59283436 4.20788802 9.61592552

Au 0.00554474 6.97093109 9.62560513

Au -1.49909859 9.44417971 9.63126788

Au -2.96536211 11.97005051 9.63737250

Au 4.48113757 4.27721658 9.62656508

Au 2.97664571 6.84359983 9.62373882

Au 1.50754487 9.44562808 9.63198998

Au -0.00471672 11.97147696 9.63849355

Au 7.37612071 4.26997277 9.63106347

Au 5.93403713 6.84533227 9.63524380

Au 4.45665374 9.40373984 9.63232868

Au 2.97507324 11.96686519 9.63645618

Au 0.00920090 5.12506599 11.58546587

C 0.00810290 7.87515499 22.60964787

C 0.00802990 4.57296599 14.50720587

C 0.00820390 8.78741599 21.62061687

C 0.00823190 9.39003099 20.55883387

C 0.00836890 9.72405399 19.24936287

C 0.00841690 9.72479799 18.03095187

C 0.00843390 9.39027499 16.72159487

C 0.00848590 8.78887899 15.65914187

C 0.00843290 7.87647199 14.67018687

C 0.00839190 6.83701699 14.01634587

C 0.00836690 5.55158299 13.53504487

C 0.00774590 3.88091399 15.51761087

C 0.00753990 3.37398299 16.76864987

C 0.00739690 3.13333099 17.96337587

C 0.00733990 3.13715999 19.31660087

C 0.00738890 3.36963899 20.51289687

C 0.00752390 3.87981099 21.76280687

C 0.00779290 4.57116499 22.77362487

C 0.00819090 5.55042899 23.74522387

C 0.00811290 6.83564899 23.26343987

Au 0.00927390 5.12557299 25.69517687

Au 0.00274052 1.69773468 27.60237065

Au 2.98578414 1.67007673 27.59851737

Au 5.92543115 1.70440831 27.59197061

Au 8.87261743 1.66556790 27.59820719

Au -1.58322102 4.21076864 27.60847080

Au -2.96112312 6.84513460 27.60933572

Au -4.44430716 9.40700337 27.59609352

Au -5.92424354 11.96472145 27.59343148

Au 1.59283436 4.20788802 27.61238422

Au 0.00554474 6.97093109 27.60270461

Au -1.49909859 9.44417971 27.59704186

Au -2.96536211 11.97005051 27.59093724

Au 4.48113757 4.27721658 27.60174466

Au 2.97664571 6.84359983 27.60457092

Au 1.50754487 9.44562808 27.59631976

Au -0.00471672 11.97147696 27.58981619

Au 7.37612071 4.26997277 27.59724627

Au 5.93403713 6.84533227 27.59306594

Au 4.45665374 9.40373984 27.59598106

Au 2.97507324 11.96686519 27.59185356

Au 0.00000000 0.00000000 29.97187974

Au 2.96230006 0.00000000 29.97187974

Au 5.92460012 0.00000000 29.97187974

Au 8.88690019 0.00000000 29.97187974

Au -1.48115003 2.56542711 29.97187974

Au -2.96230006 5.13085422 29.97187974

Au -4.44345009 7.69628132 29.97187974

Au -5.92460012 10.26170843 29.97187974

Au 1.48115003 2.56542711 29.97187974

Au 0.00000000 5.13085422 29.97187974

Au -1.48115003 7.69628132 29.97187974

Au -2.96230006 10.26170843 29.97187974

Au 4.44345009 2.56542711 29.97187974

Au 2.96230006 5.13085422 29.97187974

Au 1.48115003 7.69628132 29.97187974

Au 0.00000000 10.26170843 29.97187974

Au 7.40575016 2.56542711 29.97187974

Au 5.92460012 5.13085422 29.97187974

Au 4.44345009 7.69628132 29.97187974

Au 2.96230006 10.26170843 29.97187974

Au 1.48116491 0.85513383 32.39068974

Au 4.44346497 0.85513383 32.39068974

Au 7.40576503 0.85513383 32.39068974

Au 10.36806509 0.85513383 32.39068974

Au 0.00001488 3.42056094 32.39068974

Au -1.48113516 5.98598805 32.39068974

Au -2.96228519 8.55141515 32.39068974

Au -4.44343522 11.11684226 32.39068974

Au 2.96231494 3.42056094 32.39068974

Au 1.48116491 5.98598805 32.39068974

Au 0.00001488 8.55141515 32.39068974

Au -1.48113516 11.11684226 32.39068974

Au 5.92461500 3.42056094 32.39068974

Au 4.44346497 5.98598805 32.39068974

Au 2.96231494 8.55141515 32.39068974

Au 1.48116491 11.11684226 32.39068974

Au 8.88691506 3.42056094 32.39068974

Au 7.40576503 5.98598805 32.39068974

Au 5.92461500 8.55141515 32.39068974

Au 4.44346497 11.11684226 32.39068974

Au -0.00001483 1.71029335 34.80949974

Au 2.96228523 1.71029335 34.80949974

Au 5.92458529 1.71029335 34.80949974

Au 8.88688535 1.71029335 34.80949974

Au -1.48116486 4.27572046 34.80949974

Au -2.96231489 6.84114757 34.80949974

Au -4.44346493 9.40657467 34.80949974

Au -5.92461496 11.97200178 34.80949974

Au 1.48113520 4.27572046 34.80949974

Au -0.00001483 6.84114757 34.80949974

Au -1.48116486 9.40657467 34.80949974

Au -2.96231489 11.97200178 34.80949974

Au 4.44343526 4.27572046 34.80949974

Au 2.96228523 6.84114757 34.80949974

Au 1.48113520 9.40657467 34.80949974

Au -0.00001483 11.97200178 34.80949974

Au 7.40573532 4.27572046 34.80949974

Au 5.92458529 6.84114757 34.80949974

Au 4.44343526 9.40657467 34.80949974

Au 2.96228523 11.97200178 34.80949974

Au 0.00000000 0.00000000 37.22830974

Au 2.96230006 0.00000000 37.22830974

Au 5.92460012 0.00000000 37.22830974

Au 8.88690019 0.00000000 37.22830974

Au -1.48115003 2.56542711 37.22830974

Au -2.96230006 5.13085422 37.22830974

Au -4.44345009 7.69628132 37.22830974

Au -5.92460012 10.26170843 37.22830974

Au 1.48115003 2.56542711 37.22830974

Au 0.00000000 5.13085422 37.22830974

Au -1.48115003 7.69628132 37.22830974

Au -2.96230006 10.26170843 37.22830974

Au 4.44345009 2.56542711 37.22830974

Au 2.96230006 5.13085422 37.22830974

Au 1.48115003 7.69628132 37.22830974

Au 0.00000000 10.26170843 37.22830974

Au 7.40575016 2.56542711 37.22830974

Au 5.92460012 5.13085422 37.22830974

Au 4.44345009 7.69628132 37.22830974

Au 2.96230006 10.26170843 37.22830974

TV 11.84920025 0.00000000 0.00000000

TV -7.40575016 12.82713554 0.00000000

TV 0.00000000 0.00000000 39.64711974
